# Economic and health impacts of bovine and zoonotic tuberculosis on rural Zambian communities

**DOI:** 10.1371/journal.pgph.0005572

**Published:** 2026-06-22

**Authors:** Anthony Phiri, Mildred Zulu, Maxwell Phiri, Mulenga Malata, Sydney Kalenga, Francisco Olea-Popelka, Sydney Malama

**Affiliations:** 1 Department of Disease Control, School of Veterinary Medicine, University of Zambia, Zambia; 2 Occupational Health Services, Management Division Services, National Institute of Public Administration; 3 Department of Pathology and Microbiology, School of Medicine, University of Zambia, Zambia; 4 Department of Public Administration, School of Humanities, University of Zambia, Zambia; 5 Department of Epidemiology and Biostatistics, Schulic School of Medicine & Dentistry, University of Western Ontario, Canada; 6 Department of Biosciences and Biotechnology, School of Natural and Applied Sciences, University of Zambia, Zambia; University of Health and Allied Sciences, GHANA

## Abstract

Bovine tuberculosis is a persistent and significant challenge for cattle farmers in Zambia, especially among rural farmers. As livestock farming constitutes a crucial component of the country’s agricultural economy, the prevalence of bTB threatens not only animal health and productivity but also poses serious risks to human health, food safety, and economic stability within rural communities. A mixed-methods study was conducted in Lundazi and Monze districts of Zambia between December 2021 and June 2022, combining a cross-sectional survey of 280 respondents with qualitative insights from five focus groups and five key informant interviews. Data analysis was done using R software for quantitative data and NVivo for qualitative data. Our study indicates that cows infected with bTB experience an average decline in milk production of approximately 3.75 liters per day, translating to a substantial economic loss of approximately ZMW 10.00 per cow per day, based on an average milk price of ZMW 8.00 per liter. Reported bTB-like symptoms were significantly associated with reduced monthly income from livestock farming. Among education levels, only primary education had a significant association with bTB awareness, with higher odds of impact. Qualitative findings indicate that rural elderly individuals aged 40 and above bear a disproportionate burden of bTB’s impact on public health, likely due to livestock handling and consumption practices increasing zoonotic transmission risks. Our study reveals evidence of the significant impact of bTB on rural cattle farming, with critical implications for policy and practice. It highlights the need for appropriate interventions to address the disproportionate burden of bTB among vulnerable populations, such as older people and those with primary education. Prioritizing bTB control and prevention can minimize the economic and health impacts of the disease and promote more sustainable and resilient livestock farming systems.

## Introduction

Bovine tuberculosis (bTB) is a significant public health concern in rural Zambia, particularly in remote rural areas where livestock farming is the most important part of the agricultural economy [1]. The disease, caused by *Mycobacterium bovis*, can be transmitted to humans through the consumption of contaminated animal products or direct contact with infected animals.

Globally, zoonotic TB contributes significantly to TB burden, particularly in settings with livestock-human interaction [2,3]. In sub-Saharan Africa, *M. bovis* infections in humans have been reported, highlighting the need for integrated surveillance [4]. Zambia is among the countries with the highest burden of tuberculosis (TB) in people, with an estimated 60,000 people contracting TB annually, and approximately 15,000 succumbing to the disease every year [5]. However, in Zambia, data on *M. bovis* infection in humans are scanty [1].

The impact of bTB on human health in Zambia is substantial, with studies indicating that the disease disproportionately affects mostly the rural vulnerable populations, including those with limited access to healthcare services and those living in poverty [6]. Bovine tuberculosis triggers substantial socioeconomic costs, which are estimated at $3 billion yearly, as a result of reduced cattle productivity, milk production, and trade restrictions [5]. Reports in India revealed the prevalence of bTB, which is estimated at around 7.3%, affecting approximately 21.8 million cattle [5].

Bovine tuberculosis is a zoonotic disease that is transmissible to humans through the consumption of undercooked meat, unpasteurized dairy products, and handling with bare hands at the abattoir [7]. In the African region, about 82% of people and 85% of cattle reside in regions where bTB is prevalent [5]. Bovine tuberculosis is a significant public health concern in Zambia, more especially among the rural livestock farmers who do not practice proper hygiene, food handling, processing, and consumption practices. It was revealed that rural livestock farmers in Zambia have poor awareness of bTB transmission, with 75.3% of males and 70.3% of females expressing inadequate knowledge of the disease [5,8].

In Zambia, the prevalence of bTB has been associated with Kafue Lechwe, which acts as a wildlife reservoir host and shares grazing fields with domestic animals, mostly cattle [5]. This reveals the need for an intensive approach to control and prevent the transmission of bTB, including addressing the disease in domestic animals (mainly cattle), wildlife species (Kafue Lechwe) and humans [5].

Despite the significance of bTB being a public health concern [6], there is limited information on the impact of the disease on the rural human health population in Zambia. Numerous studies have been conducted, focusing primarily on the prevalence of bTB in cattle, with minimal attention paid to the human health implications and challenges. This continuous lack of information makes it challenging to develop effective public health strategies to control and prevent the spread of bTB [6].

Therefore, this study aimed to investigate the impact of bTB on public health in the rural population of Zambia, with a focus on identifying high-risk demographic groups and understanding the factors associated with bTB transmission. By understanding the impact of zoonotic TB on human health, policymakers and healthcare professionals can develop targeted interventions and public health strategies to mitigate the burden of the disease. Furthermore, this study contributes to a better understanding of the impact of zTB on public health in Zambia and informs evidence-based policies to protect the less privileged people in the population.

Currently in Zambia, the social determinants of TB in people, including poverty, lack of access to healthcare services, and poor living conditions, have been shown to contribute to the high burden of TB in the rural communities [6]. Additionally, the association between TB and HIV co-infection is also an imperative concern, with studies indicating that individuals with HIV are more likely to develop active TB [6,7]. Understanding the social determinants of bTB and their association with TB/HIV co-infection is crucial for developing effective public health strategies to control and prevent the spread of the disease.

In conclusion, this study aimed to contribute to a better understanding of the impact of bTB on public health in Zambia and inform the development of evidence-based policies to protect vulnerable populations. By identifying the high-risk demographic groups and understanding the factors associated with bTB transmission, policymakers and healthcare professionals can develop targeted interventions and public health strategies to mitigate the burden of the disease.

### Null and alternative hypotheses

1. Null Hypothesis (H0): There is no significant association between bTB economic and public health outcomes among rural Livestock farmers in Lundazi and Monze districts.
2. Alternative Hypothesis(H1): There is a significant association between bTB economic and public health outcomes among rural Livestock farmers in Lundazi and Monze districts, with increased risk of infection and adverse health outcomes among those with direct contact with infected animals

## Method

### Study sites

**Fig 1:**
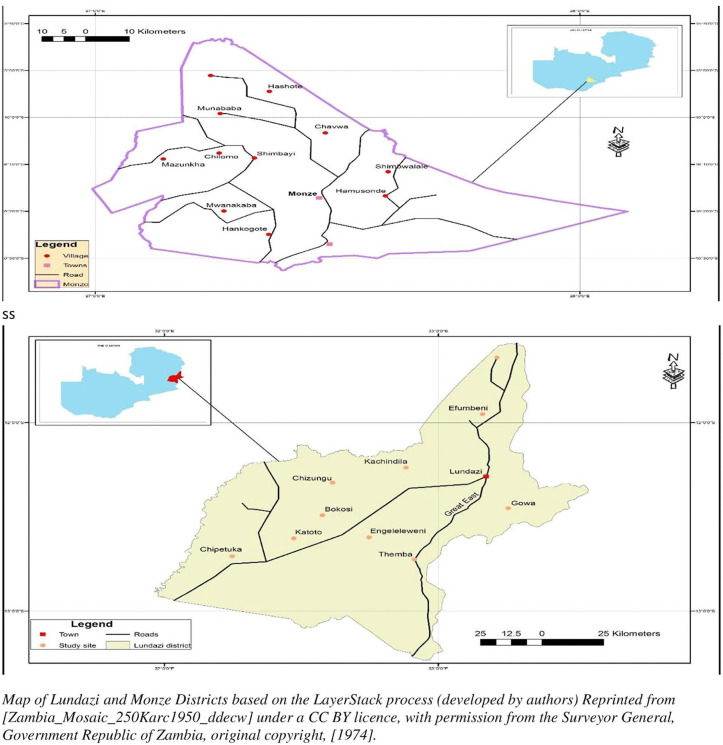
Map of Zambia showing study sites in Lundazi and Monze districts. The map was created by the Department of Geography, University of Zambia, and is reproduced with permission from the Ministry of Lands and Natural Resources, Surveyor-General’s Office, Zambia.

This research reveals critical public health concerns in districts where humans and animals coexist closely [9], and where the community practices the consumption of unpasteurized milk [10]. The study was conducted in Lundazi and Monze districts of Zambia. Lundazi is located in the eastern part of Zambia (12.2849° S, 33.1745° E), while Monze is located in the southern part of Zambia (16.2803° S, 27.4733° E) [ 11]. The selected areas have significant cattle populations. making them suitable for the study timeframe. and frequent human-animal interactions [12] (Fig 1).

### Study design

According to Kothari [13,14], a research design informs decisions concerning a research study and the arrangement of conditions for the collection and analysis of data to combine relevance to the research. To achieve the set objective, the research used a mixed-methods cross-sectional design. A key feature of mixed-methods research is its methodological pluralism or eclecticism, which frequently results in superior research compared to mono-method research [14]. Additionally, the cross-sectional design was chosen because collecting data at a single point in time is economical in terms of time, financial resources, and the nature of the study objectives [13]. (That is, it used both quantitative and qualitative methods) to collect data.

Quantitative data were gathered through a structured questionnaire administered to 280 participants, selected through multi-stage sampling (first villages, then households/workplaces). Qualitative data were collected through 5 focus group discussions (FGDs) with 104 participants, including cattle farmers, cattle traders, abattoir workers, veterinarians, and medical doctors.

### Study population and sampling

Small-scale cattle farmers in Lundazi and Monze districts were randomly selected for a cross-sectional survey. The sample included 280 respondents, comprising 208 cattle farmers, 28 cattle businessmen, 23 abattoir workers, 11 veterinary/para-veterinarians, and 10 medical doctors/clinicians. Farmers were allocated proportionally to village size, with 94 from Lundazi and 114 from Monze.

### Sample size

Initially, 373 respondents were targeted using Cochran’s formula, but due to heavy rainfall, the final sample was 280, reducing statistical power to 80% (effect size 0.3, α 0.05).

### Data collection methods and challenges

#### Interview duration.

The researcher conducted interviews for approximately 60 minutes, which is within the very much recommended time frame for in-depth interviews [13].

### Qualitative data collection methods

To ensure accuracy and validity, the researcher employed multiple methods. (a) Note-taking in a field notebook and (b) audio recording with participant consent [14] (c) Semi-structured interview guides, (d) Participant observation [15,16,17].

### Quantitative data collection methods

The researcher conducted a cross-sectional survey of 280 respondents (208 livestock farmers) [17].

### Data analysis

Data analysis involved (a) quantitative analysis using R software for statistical testing and visualization [18]; (b) thematic analysis for qualitative data, where transcripts were coded, themes identified, and patterns examined [16,19]; (c) NVivo software facilitated data management, coding, and organization for qualitative data [17]. An ordinal logistic regression model was used to evaluate the impact of bTB on monthly income. Monthly income was treated as an ordered categorical outcome variable with 5 levels based on reported Zambian Kwacha per month: 1 = ZMW 0–200, 2 = ZMW 201–400, 3 = ZMW 401–600, 4 = ZMW 601–800, and 5 = ZMW 801–1000. These cut-offs were selected to ensure adequate observations in each category. The model adjusted for education level, occupation, and gender. The proportional odds assumption was tested using the Brant test and found to be satisfied [20].

### Methodological considerations

The presence of bTB symptoms in cattle, such as ‘strong cough’ and ‘diseased animals,’ was based on self-reporting by cattle farmers, abattoir workers, and cattle businessmen, as veterinary confirmation was not feasible in this study. Income calculations were based on average milk prices reported by respondents. However, milk prices may vary seasonally and regionally, potentially affecting the accuracy of income estimates. Several factors may have influenced milk and meat yield, including co-infections with other diseases, nutritional status, and seasonal variations. While these factors were not explicitly controlled for in the analysis, the focus group discussions suggested that bTB was a major concern for cattle farmers in the study areas.

### Challenges

The researcher encountered the following challenges during data collection. (a) Time and resource constraints, (b) adverse weather, (c) inaccessible roads, € farmers’ unavailability during the rainy season [15]. Additionally, the reduced sample size limited the statistical power to 80% (assuming an effect size of 0.3, α 0.05), which may have affected the study’s ability to detect significant effects.

### Triangulation and validation

To make sure that the validity and reliability of the findings are enhanced, analytical frameworks were used to cross-check narratives from key informants with those of focus group participants, which revealed divergent or supporting views. Illustrative quotations that represented the themes were used to support the results, providing a rich and contextualized understanding of the research phenomenon.

### Ethical approval

The University of Zambia Biomedical Research Ethics Committee (UNZABREC) granted ethical approval for this study (reference number 2102–2021). Informed verbal consent was also obtained from all participants in the study areas, and the consent process was documented and witnessed by a member of the research team.

## Results

### Quantitative

Table 1 summarizes the demographic characteristics of the 280 respondents.

**Table 1:** Describe the demographic characteristics.

Variables	Category	N (%)
Gender	Male	243(86.8)
	Female	37(13.2)
Age	<30	59(21.2)
	30-40	50(17.9)
	41-50	35(12.5)
	50	136(48.6)
Education	No Education	28(10.0)
	Primary	40(14.3)
	Secondary	87(31.1)
	College	99(35)
	University	21(7.5)
	Post graduate	5(1.8)
Occupation	Cattle farmers	208(74.3)
	Human Doctor/Clinicians	10(3.6)
	Veterinary/Para-veterinarians	11(3.9)
	Abattoir Workers	23(8.2)
	Commercial business People	28(10.0)

Source: Author’s field data

### Characteristics of study subjects

The study assessed 280 cattle owned by 208 small-scale farmers from rural Zambia; 50 (17.9%) had reported bTB-like symptoms, specifically a strong cough, while 230 (82.1%) were non-diseased.

### Characteristics of study subjects

The study involved 208 small-scale cattle farmers from rural areas of Zambia. A total of 280 cattle were assessed; 50 (17.9%) had reported bTB-like symptoms, specifically a strong cough, considered potentially compatible with bTB.

### Loss of milk and meat production due to strong cough (bTB) at the household level (ZMW 18.17 per USD)

It was observed that a diseased cow (strong cough) had reduced milk production, averaging 3.75 liters/day compared to 5 liters/day for non-diseased cows (Table 2). With a milk price of ZMW8.00/liter, this translates to ZWK30.00/day (diseased) vs. ZMW40.00/day (non-diseased), resulting in an economic loss of ZWK10.00/day (Table 2).

**Table 2:** Estimated cattle milk production: Diseased vs. Non-diseased.

Measure	Animal	Production (L)	Cost (1 L/K)	Overall Cost K	Economic loss (K)
Non-Diseased	1	5	8	40.00	
Diseased	1	3.75	8	30.00	
					**10.00**

Source: Own preparation based on data from small scale cattle farmers- Lundazi Market (21 February 2022) and Bweengwe small market (17 June 2022).

Note: ‘Diseased’ refers to cattle with reported symptoms of a strong cough, potentially compatible with bTB

### Reported bTB-like symptoms significantly impacted monthly income levels

**Fig 2:**
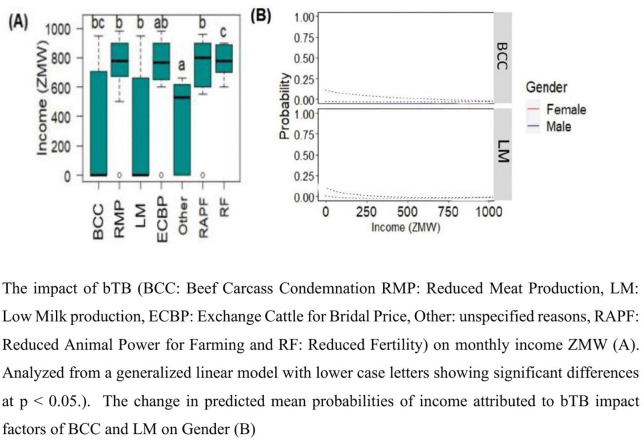
The impact of bTB (BCC: Beef Carcass Condemnation, RMP: Reduced Meat Production, LM: Low Milk production, ECBP: Exchange Cattle for Bridal Price, Other: unspecified reasons, RAPF: Reduced Animal Power for Farming, and RF: Reduced Fertility) on monthly income ZMW (A). Analyzed from a generalized linear model with lower case letters showing significant differences at p < 0.05. The change in predicted mean probabilities of income attributed to bTB impact factors of BCC and LM on Gender **(B)**.

Ordinal logistic regression was employed to examine the impact of bTB on monthly income. Income was coded as an ordered categorical variable with 5 levels: ZMW 0–200, 201–400, 401–600, 601–800, and 801–1000 per month. Results showed that reported bTB-like symptoms had a significant impact on income, suggesting increased bTB impact was linked to decreased income. Key factors were Beef Carcass Condemnation (BCC) and Low Milk production (LM), with gender differences (Fig 2). The model’s proportional odds assumption was verified and satisfied.

Among the education levels, only the primary education level was significantly impacted by bTB (p = 0.002, CI = 1.60-8.23, OR = 3.55). Other education levels were not significantly impacted by bTB (Table 3); however, the odds of being impacted by bTB were 1.24 times lower compared to other education levels. There were no significant differences in the impact of bTB on the type of occupation (p > 0.05).

**Table 3:** The impact of reported bTB-like symptoms on education levels in Zambia. The confidence intervals are indicated at 95% and the significant differences are considered at *p* < 0.05 using ordinal logistic regression.

Education level	Odds ratio	Confidence intervals	*p*-value
Primary	**3.55**	**1.60-8.23**	**0.002**
Secondary	1.28	0.53 - 3.21	0.584
College	2.08	0.95 - 4.74	0.071
University	1.32	0.43 – 4.01	0.615
Postgraduate	1.24	0.14 – 7.69	0.824

Source: Authors, field data

### Qualitative

Four main themes were yielded from the focus group discussions and interviews as follows;

- Economic Burden due to bTB
- Vulnerability of elderly people in the community
- Knowledge and Awareness Gaps Analysis
- Challenges in Controlling bTB and Prevention

### Economic burden due to bTB

It is a culture among most cattle farmers to generate income from their animals. However, when an animal has been affected by bTB, the production of milk and meat decreases drastically.

The findings indicate that the incomes of the respondents were very much affected due to the diseased animals, which could not produce the desired volume of milk.

*“I have three animals suffering from a strong cough, and the production of milk has since reduced. Initially, 1(one) animal used to produce close to 5 liters; unfortunately, to date, 1(one) animal produces only close to 3 liters of milk.* Table 1 *“[55 years old, male in Lundazi]*

‘*Most of my people in my village had experienced carcass condemnation at the abattoir, they would want to sell their animals, unfortunately, some parts like the lungs, the liver, and other organs of the cow” [Village Headman in Lundazi]*

*"I took my animal to an abattoir to be sold so that I can generate money for my firstborn child’s school fees. However, at an abattoir, some parts like the lungs* *and organs of the digestive system were condemned, such that I could not gain enough money as I expected."* [49 years old, male in Monze].

*“I could not send my two children to high school because the money that I used to generate through the sales of milk had additionally reduced, and we could not afford three meals a day. As we used to, he lasts seven years. After a good sale of milk at our local market” [35 years old, female in Monze]*

### Vulnerability of elderly people in the community

Our findings also revealed that elderly people who have been in the rearing and selling of milk and have close contact with abattoir workers have been deeply affected by the disease.

*"I have been handling and selling meat, milk, and meat products since I was young, and I have always consumed unpasteurized milk. However, two years ago, I fell ill with a fever and was referred to the main hospital in Lusaka. After some tests, I was diagnosed with tuberculosis. The doctor asked about my occupation, and I told him I’m a cattle farmer who sells milk. He advised me, at 63, to let my children or younger people handle the milk sales, as my immune system is weak due to age and I’m more susceptible to zoonotic diseases like TB." [63 years old, male in Lundazi]*

*“I grew up feeding on unpasteurized milk and on meat that was not fully cooked. I would consume meat that has not been fully prepared from beer-drinking places.” [38 years old,female in Monze]*

### Knowledge and awareness gaps analysis

Some respondents were not aware that Bovine Tuberculosis affects human health as well. They thought bTB is a disease that belongs to animals and cannot be transmitted to human beings.

*‘’I now come to realize that bovine tuberculosis (bTB) can be easily transmitted to humans through close contact with infected animals, handling meat and meat products, consuming undercooked meat, and drinking unpasteurized milk.” [38 years old, male in Monze]*

*” In my village,* my *people like the traditional way of treating diseases, which may contribute to the spread of bTB, and potential opportunities for modifying these practices.” [Village Headman in Lundazi]*

*“All along, I have been using traditional medicine to treat my animals when they are sick. I did not know that feverishness, loss of weight, loss of appetite, and prolonged cough in animals are the clinical signs of zoonotic disease like bTB”. [37 years old, male in Lundazi]*

*“I used to think that the only way to control and eliminate this disease, which affects the low production of milk and meat in our animals, is to have animals vaccinated.”[58 years old, female in Lundazi]*

Some respondents also highlight limited information about bTB and a lack of resources:

*“Regrettably ………… I have never received any piece of guidance or information about bTB from either human doctors or veterinary Doctors, specifically on how to effectively treat or control the disease. As a result, when my animals fall ill, I have been forced to rely on my limited knowledge to administer treatment without any professional prescription or guidance. Unknowingly to me, the medications I administer to my animals may be causing more harm than good, potentially leading to long-term suffering and complications for the animals.” [27 years old, male in Monze]*

*‘’In my village, we do not seem to have access to testing or diagnostic services for bTB, hence there is an impact of the disease” [38 years old, male in Lundazi]*

### Challenges in controlling bTB and prevention

Our qualitative data revealed potential challenges in controlling and preventing bTB among cattle farmers in Zambia, including limited access to resources, knowledge, and infrastructure. Therefore, by understanding these challenges, targeted interventions and support programs can be developed to address these issues and improve bTB control and prevention. Below are some of the responses:

*“I have never seen an effort by the government in terms of funding for bTB control programs; however, much of the funding has been channeled towards the fight against malaria." [Village Headman in Lundazi]*

*"I have been rearing cattle for more than 40 years; however, I am not aware of the best practices for handling milk, meat, and meat products." [61 years old, female in Lundazi]*

*“We have been consuming unpasteurized milk and undercooked meat; we do not see why we should change our lifestyle now. “ [40 years old, male in Monze]*

*“Some farmers are very skeptical about the existence risk of bTB in humans, hence they don’t take precautions.” [Village Headman in Monze]*

*“In our villages, there is a serious lack of proper facilities, which makes it hard to maintain hygiene and prevent the spread of bTB.” (37 years old, Male in Lundazi)*

## Discussion

### Milk production and household income loss

Our study found that cows with reported bTB-like symptoms produced 3.75 L/day compared to 5 L/day from non-diseased cows, representing an estimated revenue loss of ZMW 10.00 per cow per day for smallholder farmers in Lundazi and Monze. This direct quantification of daily household loss extends previous work from Ethiopia, where Tschopp et al. reported a 12% reduction in milk yield among pastoral herds with confirmed bTB but did not translate losses to household income. Similarly, Ameni et al. documented lower productivity in Holstein-Friesian versus zebu breeds under field conditions, though their focus was breed susceptibility rather than farmer income. In contrast, Barnes et al. observed only marginal reductions in milk yield among bTB-positive dairy cattle in intensive UK systems. The discrepancy likely reflects differences in production intensity, clinical stage, and breed, suggesting economic impacts are greatest in low-input smallholder systems where symptomatic animals remain in production [21–23].

### Gendered economic burden

Reported bTB-like symptoms were significantly associated with reduced monthly income in our study, with Beef Carcass Condemnation and low milk production identified as key drivers. The probability of income reduction was higher for males, consistent with qualitative reports that men dominate cattle sales and abattoir transactions in the study areas. This male-specific burden aligns with Kankya et al. in Uganda, who found that men involved in cattle trading had significantly higher odds of bTB-related financial loss due to carcass condemnation. However, gendered impacts vary by context: Mosalagae et al. reported that in Botswana, women bore greater economic risk because they managed daily milking and household milk processing, while men handled occasional sales. These comparisons indicate that the gender most affected depends on local division of livestock-related labour [24,25].

### Education as a determinant of vulnerability

Ordinal logistic regression showed that only primary education was significantly associated with bTB impact (OR=3.55, 95% CI = 1.60–8.23, p = 0.002); secondary, college, and university levels were not significant. This suggests that minimal formal education limits awareness of zoonotic transmission and prevention. Our finding is consistent with Mukherjee et al. in India, who reported that farmers with education below 5th grade had 3.1 times lower knowledge scores on bTB zoonosis. It also agrees with Tigre et al. in Ethiopia, who found that completion of primary school was the threshold above which adoption of milk boiling and other preventive practices increased significantly. Across African and South Asian smallholder systems, primary education appears to be a consistent modifier of bTB-related risk [26].

### Age-related vulnerability

Qualitative data indicated that respondents aged ≥40 years reported greater vulnerability to bTB impacts, citing lifelong consumption of unpasteurized milk, regular handling of carcasses, and limited contact with veterinary services. This pattern matches Gumi et al. in Ethiopia, who reported higher M. bovis seroprevalence among pastoralists >45 years due to cumulative occupational exposure. However, it differs from UK data reviewed by Broughan et al., where bTB reactors were concentrated in prime-age cattle and human cases showed no age bias, likely reflecting universal pasteurization and different occupational roles. We acknowledge that without bacteriological confirmation our study cannot determine true age-specific M. bovis prevalence, and memory decline in elderly respondents may have influenced recall of practices [8,27].

### Implications for one health policy

The vulnerability of rural populations identified here reinforces the need for a One Health approach to bTB surveillance and control. This aligns with WHO, which designates bTB a neglected zoonosis requiring multisectoral action, and with the FAO-WOAH-WHO tripartite, which prioritizes bTB for integrated surveillance at the human-animal interface. Our data extend these frameworks by identifying specific intervention targets for Zambia, farmers with only primary education, and those aged ≥40 years.

### Recent amendment

In 2023, Zambia’s Ministry of Fisheries and Livestock adopted revised guidelines for bTB testing at slaughter, increasing compensation thresholds for condemned carcasses. Our findings on male-specific income loss from BCC suggest these guidelines should be paired with farmer education to ensure uptake [20,28,29].

### Methodological considerations and recent context

A key limitation is that ‘diseased’ status was based on farmer-reported strong cough rather than veterinary or laboratory confirmation. Thus, our economic estimates reflect losses associated with reported bTB-like symptoms, not confirmed bTB. This syndromic approach is similar to Katale et al. in Tanzania, who used farmer reports when diagnostic testing was infeasible, but contrasts with abattoir studies like Asseged et al., which relied on detailed post-mortem inspection and culture. Income estimates also used average milk prices reported by respondents and may not capture seasonal variation, a limitation also noted by Srinivasan et al. in their economic analysis of bTB in India. Recent amendment: The 2024 WOAH Terrestrial Animal Health Code update emphasizes syndromic surveillance in low-resource settings as a complement to test-and-slaughter. Our study provides baseline data for such syndromic approaches in Zambia’s smallholder sector [30–32,33].

The study’s findings provide district-level evidence that reported bTB-like symptoms are associated with measurable daily milk losses and reduced monthly income in Zambia, with disproportionate burden among males, farmers with primary education, and those aged ≥40 years. By distinguishing these household impacts from existing literature and incorporating recent policy changes, the findings can inform targeted education, compensation, and surveillance programs.

### Recommendations

i Implement targeted education and awareness programs for rural livestock farmers, focusing on bTB transmission, control, and prevention measures.
ii Enhance access to veterinary services in rural areas, providing regular guidance and support to farmers.
iii Conduct regular testing and vaccination programs, as current efforts are insufficient to minimize bTB prevalence; further research is needed to develop effective disease control measures.
iv Provide comprehensive economic support and compensation to farmers experiencing bTB-related losses, mitigating the financial impact.
v Target interventions towards rural elderly livestock farmers, improving awareness and knowledge of bTB control measures.
vi Further research is needed to assess the economic impact of bTB, informing policy and decision-making for effective control strategies.

### Limitations

The study findings only focused on bTB; however, other zoonotic diseases or risk factors may also be present in the study area, which could influence the findings. Additionally, the study’s cross-sectional design may not capture the dynamic nature of bTB transmission and its public health impact over time. The study may have underestimated the real impact of bTB on public health because of a lack of awareness about the disease. Lastly, the study was conducted in specific districts, Lundazi and Monze, respectively, which may not be representative of the entire country or region.

## Supporting information

S1 Data Dataset for bovine tuberculosis study in Lundazi and Monze districts, Zambia. Contains survey responses from 280 participants, including variables on cattle milk production, reported bTB-like symptoms, household income, education level, occupation, age, and gender collected between December 2021 and June 2022.
(XLSX)
