# Supplementary material for: Economic and health impacts of bovine and zoonotic tuberculosis on rural Zambian communities
Source: PLOS Glob Public Health. 2026 Jun 22;6(6):e0005572. doi: 10.1371/journal.pgph.0005572 (PMC13286274; doi:10.1371/journal.pgph.0005572)
Supplement: Attachment — Submitted filename: Review Report_PLOS GPH_Zambia bTB_4.12.2025.pdf [file pgph.0005572.s002.pdf]

PLOS Global Public Health

ECONOMIC AND HEALTH IMPACTS OF BOVINE TUBERCULOSIS ON RURAL ZAMBIAN COMMUNITIES

General Assessment

This manuscript addresses a relevant and timely topic, exploring the economic and health impacts of bovine tuberculosis (bTB) on rural communities in Zambia through a mixed-methods approach. The work is valuable and provides important insights into the socioeconomic vulnerabilities associated with bTB. However, several areas require clarification and strengthening to enhance the scientific robustness and public health relevance of the study.

Major Comments

1. **Missing epidemiological context on zoonotic TB in humans**

The manuscript discusses the public health implications of bTB but does not provide available data on ***M. bovis* infection prevalence in humans** at:

- national level,
- district level (Lundazi and Monze),
- or from comparable regions in sub-Saharan Africa.

To address this gap, please consider integrating key global references on zoonotic TB, such as:

- WHO (2017). *Roadmap for Zoonotic tuberculosis* https://www.who.int/publications/i/item/9789241513043
- Olea-Popelka, F., & Fujiwara, P. I. (2018). Building a Multi-Institutional and Interdisciplinary Team to Develop a Zoonotic Tuberculosis Roadmap. Frontiers in Public Health, 6, 167. https://www.frontiersin.org/articles/10.3389/fpubh.2018.00167/full

Including these references will help contextualize the burden of zoonotic TB and strengthen the public health discussion and better support conclusions.

2. **Public health implications are underdeveloped**

While the economic impact of bTB is well described, the public health dimension is comparatively limited.

The manuscript would benefit from:

- more explicit discussion of **zoonotic risks for different demographic groups**,
- potential barriers to diagnosis and reporting of *M. bovis* in rural healthcare settings,
- implications for One Health surveillance.

This would provide a more balanced interpretation aligned with the study objectives.

3. **Limited comparison with existing literature**

The discussion currently focuses mainly on East and Southern Africa. It would be helpful to cite global and regional reviews addressing the **wildlife–livestock–human interface**, which is central to bTB epidemiology in Zambia.

Please consider adding:

De GARINE-WICHATITSKY M, CARON A, KOCK R, et al. 2013 (Cambridge): *A review of bovine tuberculosis at the wildlife–livestock–human interface in sub-Saharan Africa* https://www.cambridge.org/core/journals/epidemiology-and-infection/article/review-of-bovine-tuberculosis-at-the-wildlifelivestockhuman-interface-in-subsaharan-africa/19D207B4D88531AB03A96FEF7BF6F95E

Munyeme et al. (2011). *A Review of Bovine Tuberculosis in the Kafue Basin Ecosystem* https://pmc.ncbi.nlm.nih.gov/articles/PMC3087610/

These references are particularly relevant given **the role of Kafue lechwe** as a reservoir species and the importance of studying disease dynamics at the domestic–wildlife–human interface.

4. **Interpretation of increased vulnerability in elderly respondents**

The interpretation that elderly individuals are more affected because of reduced immunity and lower awareness requires careful qualification. While it is plausible that older adults may be more likely to progress to clinical disease due to immunosenescence, the study did not include any diagnostic testing for *M. bovis* infection in humans.

Without diagnostic data, such as tuberculin skin testing (e.g., the Mantoux test), interferon-gamma release assays, or microbiological confirmation, the study cannot infer the **true prevalence of mycobacteria infection** across age groups.

It is important to acknowledge that younger individuals may have similar or even higher infection rates but remain asymptomatic due to a more effective immune response.

Thus, the distinction between:

- infection prevalence (which requires diagnostic testing), and
- clinical disease expression (more common in immunosuppressed or elderly individuals)

should be clearly stated to avoid overinterpretation of the findings.

5. **Methodological clarifications required**

Several methodological details require further explanation:

- Clarify whether “strong cough” and “diseased animals” were self-reported or confirmed by veterinary staff.
- Consider discussing potential confounders affecting milk/meat yield (other diseases, nutrition, seasonality).
- Income calculations assume fixed milk prices; please comment on possible seasonal or regional/geographic price variability.

Addressing these points will improve methodological transparency.

Minor Comments

1. Some sections require language editing to improve clarity and flow.

2. Figures and tables would benefit from clearer captions and more detailed descriptions.

3. A brief description of cattle management systems in Lundazi and Monze would provide useful context for interpreting transmission risks.

4. The Discussion could better highlight the value added by the mixed-methods approach.

5. You may consider revising the reference list, as several entries appear duplicated. Specifically, the following references are listed more than once:

- Demetriou 2020 (Refs. 23 and 26)
- Oloya et al. (Refs. 29 and 37)
- Ameni et al. (Refs. 30, 38, and 45)
- Kansiime et al. (Refs. 28 and 36)
- Kazwala et al. (Refs. 32 and 39)
- Mfinanga et al. (Refs. 33 and 40)
- Duguma et al. (Refs. 34 and 41)
- Kassa et al. (Refs. 35 and 42)

Additionally, two reviews by Tschopp (Refs. 18 and 24) appear very similar and may need verification to confirm they are distinct publications.

A careful review of the reference list is recommended to avoid redundancy and improve clarity.

Overall Recommendation

The manuscript provides useful insights but requires substantial revisions. Strengthening the epidemiological context, integrating key references, and refining the public health discussion will significantly improve the impact and clarity of the work and enhance its contribution to *PLOS Global Public Health*.
