# Supplementary material for: Economic and health impacts of bovine and zoonotic tuberculosis on rural Zambian communities
Source: PLOS Glob Public Health. 2026 Jun 22;6(6):e0005572. doi: 10.1371/journal.pgph.0005572 (PMC13286274; doi:10.1371/journal.pgph.0005572)
Supplement: Attachment — Submitted filename: PLOS GPH_Report 3.docx.pdf [file pgph.0005572.s005.pdf]

PLOS Global Public Health

ECONOMIC AND HEALTH IMPACTS OF BOVINE TUBERCULOSIS ON RURAL ZAMBIAN COMMUNITIES

1. **Major comment – Case definition of bovine tuberculosis**

As already noted in my previous report, the manuscript discusses the impact of bovine tuberculosis (bTB) on cattle production and household income. Following the clarification now included in the Methods section, **the presence of bTB in cattle appears to be based on self-reported symptoms (e.g., "strong cough") provided by farmers and other participants**, without veterinary confirmation or diagnostic testing. Since respiratory symptoms alone are not specific to bTB, this approach may lead to **potential misclassification of cases**. I therefore recommend clarifying throughout the manuscript that the study refers **to reported disease signs potentially compatible with bTB**, rather than confirmed bTB infections, to avoid possible over-interpretation of the findings.

In addition, the Results section reports that 50 out of 280 cattle (17.9%) showed signs of "strong cough", which is interpreted as evidence of bTB impact. Given that this estimate is based on self-reported symptoms rather than diagnostic confirmation, this figure should be interpreted more cautiously. It would be helpful to clarify that this proportion **reflects reported respiratory symptoms potentially compatible with disease, and therefore the perceived presence and impact of disease among farmers, rather than confirmed bTB prevalence**, which would strengthen the epidemiological interpretation of the results.

2. **Major comment – Statistical modelling**

The description and interpretation of the ordinal logistic regression model require further clarification. In particular, the manuscript does not clearly define the ordinal outcome variable used in the model, nor how monthly income was categorized to justify the use of an ordinal regression approach. In addition, the reported regression results appear inconsistent (e.g., OR = 0.99 with CI = 0.997–0.998), and the interpretation of the odds ratio is difficult to follow. I recommend verifying the regression outputs and clearly describing the model specification, variables included, and the interpretation of the results. In addition, since ordinal logistic regression relies on the proportional odds assumption, it would be useful to indicate whether this assumption was assessed. A careful review of the statistical analysis would help ensure that the model specification and interpretation are appropriate.

3. **Minor Comment – Methods structure**

The Methods section has been expanded in the revised manuscript, which improves transparency. However, some subsections partially overlap (e.g., *Sample size*, *Selection of participants*, and *Study population and sampling strategy*). Consider reorganizing these sections to improve readability and reduce redundancy.

4. **Minor Comment – Economic loss estimates**

The description of milk production losses requires clarification. The text indicates that a diseased cow shows "a reduction in milk production of close to 3.75 liters", whereas Table 2 suggests that milk production decreases from 5 L to 3.75 L (i.e., a reduction of 1.25 L). Please ensure consistency between the text and the table and clarify the calculation of the reported economic losses.

5. **Minor Comment – Language and typographical issues**

The manuscript is generally understandable; however, some minor language issues and typographical errors remain (e.g., “These areas boast of substantial cattle populations”, “very much recommended time frame”, and “1medical practitioners”). A careful language editing would improve clarity and overall readability.
