# Supplementary material for: Economic and health impacts of bovine and zoonotic tuberculosis on rural Zambian communities
Source: PLOS Glob Public Health. 2026 Jun 22;6(6):e0005572. doi: 10.1371/journal.pgph.0005572 (PMC13286274; doi:10.1371/journal.pgph.0005572)
Supplement: Attachment — Submitted filename: RESPONSE TO THE REVIEWERS.docx [file pgph.0005572.s003.docx]

**RESPONSE TO THE REVIEWERS - REVIEWERS 1 AND 2**

Dear Authors,
Both reviewers agree that your manuscript addresses a relevant and timely topic by examining the economic and health impacts of bovine tuberculosis (bTB) on rural communities in Zambia using a mixed-methods approach. The study is valuable and offers important insights into the socioeconomic vulnerabilities associated with bTB. However, several aspects of the manuscript require further clarification and strengthening to improve its scientific rigor and enhance its public health relevance. We therefore invite you to carefully consider and address the reviewers’ comments to strengthen the manuscript and enhance its suitability for publication.


[Note: HTML markup is below. Please do not edit.]

Reviewer's Responses to Questions

**Comments to the Author**

1. Does this manuscript meet PLOS Global Public Health’s [publication criteria](https://track.editorialmanager.com/CL0/https:%2F%2Fjournals.plos.org%2Fglobalpublichealth%2Fs%2Fcriteria-for-publication/1/010f019be9586502-ee673f0d-e340-48e3-89da-148ba57a0005-000000/c6lcn_DeDZLzQ0xXXlmKIeOp7__ZXXei94TcScVFKKg=247)? Is the manuscript technically sound, and do the data support the conclusions? The manuscript must describe methodologically and ethically rigorous research with conclusions that are appropriately drawn based on the data presented.

Reviewer #1: Partly

2. Has the statistical analysis been performed appropriately and rigorously?

Reviewer #1: I don't know

**THIS HAS BEEN FULLY CORRECTED**

3.  Have the authors made all data underlying the findings in their manuscript fully available (please refer to the Data Availability Statement at the start of the manuscript PDF file)?

The [PLOS Data policy](https://track.editorialmanager.com/CL0/https:%2F%2Fjournals.plos.org%2Fglobalpublichealth%2Fs%2Fdata-availability/1/010f019be9586502-ee673f0d-e340-48e3-89da-148ba57a0005-000000/cTFmiL6Ig-JF_u2fydXvTuwNVONelfxllw9G5ND-rbE=247) requires authors to make all data underlying the findings described in their manuscript fully available without restriction, with rare exception. The data should be provided as part of the manuscript or its supporting information, or deposited to a public repository. For example, in addition to summary statistics, the data points behind means, medians and variance measures should be available. If there are restrictions on publicly sharing data—e.g. participant privacy or use of data from a third party—those must be specified.

Reviewer #1: No

**DATA SET HAS BEEN ADDED**

4.  Is the manuscript presented in an intelligible fashion and written in standard English?

PLOS Global Public Health does not copyedit accepted manuscripts, so the language in submitted articles must be clear, correct, and unambiguous. Any typographical or grammatical errors should be corrected at revision, so please note any specific errors here.

Reviewer #1: No

**WE HAVE CURRENTLY ENGAGE EXPART IN ENGLISH AND THIS ISSUE HAS BEEN SORTED OUT**

 Review Comments to the Author

Please use the space provided to explain your answers to the questions above. You may also include additional comments for the author, including concerns about dual publication, research ethics, or publication ethics. (Please upload your review as an attachment if it exceeds 20,000 characters)

Reviewer #1: PLOS Global Public Health
ECONOMIC AND HEALTH IMPACTS OF BOVINE TUBERCULOSIS ON RURAL ZAMBIAN COMMUNITIES

**General Assessment**
This manuscript addresses a relevant and timely topic, exploring the economic and health impacts of bovine tuberculosis (bTB) on rural communities in Zambia through a mixed-methods approach. The work is valuable and provides important insights into the socioeconomic vulnerabilities associated with bTB. However, several areas require clarification and strengthening to enhance the scientific robustness and public health relevance of the study.

**Major Comments**

1.Missing epidemiological context on zoonotic TB in humans
The manuscript discusses the public health implications of bTB but does not provide available data on M. bovis infection prevalence in humans at:
• national level,
• district level (Lundazi and Monze),
• or from comparable regions in sub-Saharan Africa.
To address this gap, please consider integrating key global references on zoonotic TB, such as:
• WHO (2017). Roadmap for Zoonotic tuberculosis <https://www.who.int/publications/i/item/9789241513043>
• Olea-Popelka, F., & Fujiwara, P. I. (2018). Building a Multi-Institutional and Interdisciplinary Team to Develop a Zoonotic Tuberculosis Roadmap. Frontiers in Public Health, 6, 167.
<https://www.frontiersin.org/articles/10.3389/fpubh.2018.00167/full>
Including these references will help contextualize the burden of zoonotic TB and strengthen the public health discussion and better support conclusions.\

**DONE**

2. Public health implications are underdeveloped
While the economic impact of bTB is well described, the public health dimension is comparatively limited.
The manuscript would benefit from:
• more explicit discussion of zoonotic risks for different demographic groups,
• potential barriers to diagnosis and reporting of M. bovis in rural healthcare settings,
• implications for One Health surveillance.
This would provide a more balanced interpretation aligned with the study objectives.

**DONE**

1. Limited comparison with existing literature
   The discussion currently focuses mainly on East and Southern Africa. It would be helpful to cite global and regional reviews addressing the wildlife–livestock–human interface, which is central to bTB epidemiology in Zambia.
   Please consider adding:
   De GARINE-WICHATITSKY M, CARON A, KOCK R, et al. 2013 (Cambridge): A review of bovine tuberculosis at the wildlife–livestock–human interface in sub-Saharan Africa <https://www.cambridge.org/core/journals/epidemiology-and-infection/article/review-of-bovine-tuberculosis-at-the-wildlifelivestockhuman-interface-in-subsaharan-africa/19D207B4D88531AB03A96FEF7BF6F95E>
   Munyeme et al. (2011). A Review of Bovine Tuberculosis in the Kafue Basin Ecosystem <https://pmc.ncbi.nlm.nih.gov/articles/PMC3087610/>
   These references are particularly relevant given the role of Kafue lechwe as a reservoir species and the importance of studying disease dynamics at the domestic–wildlife–human interface.

**DONE**

2. Interpretation of increased vulnerability in elderly respondents
   The interpretation that elderly individuals are more affected because of reduced immunity and lower awareness requires careful qualification. While it is plausible that older adults may be more likely to progress to clinical disease due to immunosenescence, the study did not include any diagnostic testing for M. bovis infection in humans.
   Without diagnostic data, such as tuberculin skin testing (e.g., the Mantoux test), interferon-gamma release assays, or microbiological confirmation, the study cannot infer the true prevalence of mycobacteria infection across age groups.
   It is important to acknowledge that younger individuals may have similar or even higher infection rates but remain asymptomatic due to a more effective immune response.
   Thus, the distinction between:
   • infection prevalence (which requires diagnostic testing), and
   • clinical disease expression (more common in immunosuppressed or elderly individuals)
   should be clearly stated to avoid overinterpretation of the findings.

**DONE**

3. Methodological clarifications required
   Several methodological details require further explanation:
   • Clarify whether “strong cough” and “diseased animals” were self-reported or confirmed by veterinary staff.
   • Consider discussing potential confounders affecting milk/meat yield (other diseases, nutrition, seasonality).
   • Income calculations assume fixed milk prices; please comment on possible seasonal or regional/geographic price variability.
   Addressing these points will improve methodological transparency.

**DONE**

**Minor Comments**
1. Some sections require language editing to improve clarity and flow.**DONE**
2. Figures and tables would benefit from clearer captions and more detailed descriptions**.DONE**
3. A brief description of cattle management systems in Lundazi and Monze would provide useful context for interpreting transmission risks. **DONE**
4. The Discussion could better highlight the value added by the mixed-methods approach.**DONE**
5. You may consider revising the reference list, as several entries appear duplicated. Specifically, the following references are listed more than once:
− Demetriou 2020 (Refs. 23 and 26)
− Oloya et al. (Refs. 29 and 37)
− Ameni et al. (Refs. 30, 38, and 45)
− Kansiime et al. (Refs. 28 and 36)
− Kazwala et al. (Refs. 32 and 39)
− Mfinanga et al. (Refs. 33 and 40)
− Duguma et al. (Refs. 34 and 41)
− Kassa et al. (Refs. 35 and 42)
Additionally, two reviews by Tschopp (Refs. 18 and 24) appear very similar and may need verification to confirm they are distinct publications.
A careful review of the reference list is recommended to avoid redundancy and improve clarity. **DONE**

**Overall Recommendation**
The manuscript provides useful insights but requires substantial revisions. Strengthening the epidemiological context, integrating key references, and refining the public health discussion will significantly improve the impact and clarity of the work and enhance its contribution to PLOS Global Public Health.

**Reviewer 2**

PLOS Global Public Health
ECONOMIC AND HEALTH IMPACTS OF BOVINE TUBERCULOSIS ON RURAL ZAMBIAN COMMUNITIES

The introduction lacks information on the prevalence of bTB in humans.

**DONE**

*Bovine tuberculosis is a significant public health concern in Zambia, more especially among the rural livestock farmers who do not practice hygiene:* Has it been established that unhygienic practices lead to bTB? If not, then this sentence will require some revision.

**DONE**

*Numerous studies have been conducted, focusing primarily on the prevalence of bTB in cattle, with minimal attention paid to the human health implications and challenges*: In spite of this statement, not much has been said regarding the implications of bTB on human health.

CORRECTED

**Methods**

The sample design should be changed to a study design. The content for the study design focused more on what mixed method designs are all about instead of indicating what was done for this study.

**DONE**

Information on sample size estimation was missing in the methods section, as well as how participants were recruited into the study.

**DONE**

**Study Population and Sampling Strategy**

The authors did not make mention of the category of farmers involved in the focus group discussion and the key informant interviews.

**DONE**

**Data Analysis Tools**

Information on the analysis was very scanty. The authors did not describe in detail how the data has been analysed.

**DONE**

**Results**

Even though the authors estimated 280 cattle farmers for the survey, there was no indication in the results whether all 280 farmers participated in the interviews or not.

## **DONE This has been mentioned and covered in the sample size**

The quantitative results did not present Table 1 to describe the demographic characteristics of the cattle farmers. Results presented for the quantitative part appear as if only secondary data was used. If that is not the case, the authors should kindly present much more detailed results than what is presented currently.

**DONE**

*It was observed that a diseased cow (strong cough) will incur a reduction in milk production of an average of close to 3.75 liters.* This is not an observation made, hence I suggest revising it as reported. But before this sentence, it is advisable to describe the characteristics of the study subjects. Also, the authors should indicate what proportion of cows were deceased (experienced cough) and non-diseased.

Table 1 has no clear title. If Table 1 compared the estimated milk production between diseased and non-diseased animals, this should be made clear.

*Quantitative and qualitative data were taken from Lundazi and Bweengwe's small market*: Here, it is not clear if these were farmers or not.

**DONE**

*Figure 2*: The title could not be separated from the text.

The change in predicted mean probabilities of income attributed to bTB impact factors of BCC and LM on Gender (B): This sentence is missing a full stop.

*Among the education levels, only the primary education level was significantly impacted by bTB (p = 0.002, CI = 1.60-8.23, OR = 3.55):* This will need some correction of grammar.

**DONE**

**Economic Burden due to bTB**

*However, when an animal has been affected by bTB. The production of milk and meat decreases drastically:* A comma has been omitted here.

**DONE**

**Discussion**

Probably the topic should have been the economic and health impact of bTB on milk production and meat yield in rural Zambian communities.

THE TOPIC HAS BEEN CHANGED TO **ECONOMIC AND HEALTH IMPACTS OF BOVINE AND ZOONOTIC TUBERCULOSIS ON RURAL ZAMBIAN COMMUNITIES**

Recommendations are not targeted to specific individuals or organizations who are required to carry them out. Recommendation number 3 does not look complete.

**DONE**

The authors should indicate what they did not do right, which is why they are recommending a similar study for further research, as in recommendation number 6.

**DONE**
