# Supplementary material for: Economic and health impacts of bovine and zoonotic tuberculosis on rural Zambian communities
Source: PLOS Glob Public Health. 2026 Jun 22;6(6):e0005572. doi: 10.1371/journal.pgph.0005572 (PMC13286274; doi:10.1371/journal.pgph.0005572)
Supplement: Attachment — Submitted filename: REBUTTAL LETTER 22APRIL26.docx [file pgph.0005572.s007.docx]

REBUTTAL LETTER

Reviewer Comment: Please explicitly report the income categories (including cut-off values) used to define the ordered outcome variable in the ordinal logistic regression model.

Response: We have now explicitly defined the income categories as ZMW 0–200, 201–400, 401–600, 601–800, and 801–1000. This definition has been added to the Methods section under Data Analysis, page 7, and clarified in the Results section, page 13, in the tracked changes manuscript.

Reviewer Comment: A final round of careful language editing is needed to address minor grammatical issues and ensure consistency in terminology (e.g., consistent use of “reported bTB-like symptoms” where appropriate, and removal of duplicated section headings).

Response: We have conducted a full language edit. Terminology has been standardized to “reported bTB-like symptoms” throughout when referring to farmer-reported cases. The duplicated “Characteristics of Study Subjects” heading and repeated sentence were deleted. Currency is now consistently ZMW. Minor grammatical errors were corrected.
