# Supplementary material for: Economic and health impacts of bovine and zoonotic tuberculosis on rural Zambian communities
Source: PLOS Glob Public Health. 2026 Jun 22;6(6):e0005572. doi: 10.1371/journal.pgph.0005572 (PMC13286274; doi:10.1371/journal.pgph.0005572)
Supplement: Peer review history

# Peer review: Decision Letter 0

**DOI:** 10.1371/journal.pgph.0005572.r001

**Authors:** Mavis Pearl Kwabla

23 Jan 2026

PGPH-D-25-03539

ECONOMIC AND HEALTH IMPACTS OF BOVINE TUBERCULOSIS ON RURAL ZAMBIAN COMMUNITIES

PLOS Global Public Health

Dear Dr. Phiri Anthony

Thank you for submitting your manuscript to PLOS Global Public Health. After careful consideration, we feel that it has merit but does not fully meet PLOS Global Public Health’s publication criteria as it currently stands. Therefore, we invite you to submit a revised version of the manuscript that addresses the points raised during the review process.

Please submit your revised manuscript by Mar 09 2026 11:59PM. If you will need more time than this to complete your revisions, please reply to this message or contact the journal office at globalpubhealth@plos.org. When you're ready to submit your revision, log on to https://www.editorialmanager.com/pgph/ and select the 'Submissions Needing Revision' folder to locate your manuscript file.

Please include the following items when submitting your revised manuscript:

- A letter that responds to each point raised by the editor and reviewer(s). You should upload this letter as a separate file labeled 'Response to Reviewers'.
- A marked-up copy of your manuscript that highlights changes made to the original version. You should upload this as a separate file labeled 'Revised Manuscript with Track Changes'.
- An unmarked version of your revised paper without tracked changes. You should upload this as a separate file labeled 'Manuscript'.

Guidelines for resubmitting your figure files are available below the reviewer comments at the end of this letter.

We look forward to receiving your revised manuscript.

Kind regards,

Mavis Pearl Kwabla, Master of Public Health

Academic Editor

PLOS Global Public Health

Journal Requirements:

1. Please provide additional details regarding participant consent. In the ethics statement in the Methods and online submission information, please ensure that you have specified (1) whether consent was informed and (2) what type you obtained (for instance, written or verbal, and if verbal, how it was documented and witnessed). If your study included minors, state whether you obtained consent from parents or guardians. If the need for consent was waived by the ethics committee, please include this information.

If you are reporting a retrospective study of medical records or archived samples, please ensure that you have discussed whether all data were fully anonymized before you accessed them and/or whether the IRB or ethics committee waived the requirement for informed consent. If patients provided informed written consent to have data from their medical records used in research, please include this information.

2. Please amend your online Financial Disclosure statement. If you did not receive any funding for this study, please simply state: “The authors received no specific funding for this work.”

3. Please update your online Competing Interests statement. If you have no competing interests to declare, please state: “The authors have declared that no competing interests exist.”

4. We note that your Data Availability Statement is currently as follows: “All data can be accessed as required”

Please confirm at this time whether or not your submission contains all raw data required to replicate the results of your study. Authors must share the “minimal data set” for their submission. PLOS defines the minimal data set to consist of the data required to replicate all study findings reported in the article, as well as related metadata and methods (https://journals.plos.org/globalpublichealth/s/data-availability#loc-minimal-data-set-definition).

For example, authors should submit the following data:

- The values behind the means, standard deviations and other measures reported;

- The values used to build graphs;

- The points extracted from images for analysis.

Authors do not need to submit their entire data set if only a portion of the data was used in the reported study.

If your submission does not contain these data, please either upload them as Supporting Information files or deposit them to a stable, public repository and provide us with the relevant URLs, DOIs, or accession numbers. For a list of recommended repositories, please see https://journals.plos.org/globalpublichealth/s/recommended-repositories.

If there are ethical or legal restrictions on sharing a de-identified data set, please explain them in detail (e.g., data contain potentially sensitive information, data are owned by a third-party organization, etc.) and who has imposed them (e.g., an ethics committee). Please also provide contact information for a data access committee, ethics committee, or other institutional body to which data requests may be sent. If data are owned by a third party, please indicate how others may request data access.

5. Please provide separate main figure files in .tif or .eps format only and remove any figures embedded in your manuscript file. Please also ensure that all files are under our size limit of 10MB. Please leave the figure captions in the manuscript.

For more information about how to convert your figure files please see our guidelines: https://journals.plos.org/globalpublichealth/s/figures

6. Some material included in your submission may be copyrighted. According to PLOS’s copyright policy, authors who use figures or other material (e.g., graphics, clipart, maps) from another author or copyright holder must demonstrate or obtain permission to publish this material under the Creative Commons Attribution 4.0 International (CC BY 4.0) License used by PLOS journals. Please closely review the details of PLOS’s copyright requirements here: PLOS Licenses and Copyright. If you need to request permissions from a copyright holder, you may use PLOS's Copyright Content Permission form.

Please respond directly to this email or email the journal office and provide any known details concerning your material's license terms and permissions required for reuse, even if you have not yet obtained copyright permissions or are unsure of your material's copyright compatibility.

Potential Copyright Issues:

Figure 1: please (a) provide a direct link to the base layer of the map (i.e., the country or region border shape) and ensure this is also included in the figure legend; and (b) provide a link to the terms of use / license information for the base layer image or shapefile. We cannot publish proprietary or copyrighted maps (e.g. Google Maps, Mapquest) and the terms of use for your map base layer must be compatible with our CC-BY 4.0 license.

Note: if you created the map in a software program like R or ArcGIS, please locate and indicate the source of the basemap shapefile onto which data has been plotted.

If your map was obtained from a copyrighted source please amend the figure so that the base map used is from an openly available source. Alternatively, please provide explicit written permission from the copyright holder granting you the right to publish the material under our CC-BY 4.0 license.

Please note that the following CC BY licenses are compatible with PLOS license: CC BY 4.0, CC BY 2.0 and CC BY 3.0, meanwhile such licenses as CC BY-ND 3.0 and others are not compatible due to additional restrictions.

If you are unsure whether you can use a map or not, please do reach out and we will be able to help you. The following websites are good examples of where you can source open access or public domain maps:

* U.S. Geological Survey (USGS) - All maps are in the public domain. (http://www.usgs.gov)

* PlaniGlobe - All maps are published under a Creative Commons license so please cite “PlaniGlobe, http://www.planiglobe.com, CC BY 2.0” in the image credit after the caption. (http://www.planiglobe.com/?lang=enl)

* Natural Earth - All maps are public domain. (http://www.naturalearthdata.com/about/terms-of-use/)

7. If the reviewer comments include a recommendation to cite specific previously published works, please review and evaluate these publications to determine whether they are relevant and should be cited. There is no requirement to cite these works unless the editor has indicated otherwise.

Additional Editor Comments (if provided):

Dear Authors,

Both reviewers agree that your manuscript addresses a relevant and timely topic by examining the economic and health impacts of bovine tuberculosis (bTB) on rural communities in Zambia using a mixed-methods approach. The study is valuable and offers important insights into the socioeconomic vulnerabilities associated with bTB. However, several aspects of the manuscript require further clarification and strengthening to improve its scientific rigor and enhance its public health relevance. We therefore invite you to carefully consider and address the reviewers’ comments to strengthen the manuscript and enhance its suitability for publication.

[Note: HTML markup is below. Please do not edit.]

Reviewers' comments:

Reviewer's Responses to Questions

**Comments to the Author**

1. Does this manuscript meet PLOS Global Public Health’s publication criteria? Is the manuscript technically sound, and do the data support the conclusions? The manuscript must describe methodologically and ethically rigorous research with conclusions that are appropriately drawn based on the data presented.

Reviewer #1: Partly

**********

2. Has the statistical analysis been performed appropriately and rigorously?

Reviewer #1: I don't know

**********

3. Have the authors made all data underlying the findings in their manuscript fully available (please refer to the Data Availability Statement at the start of the manuscript PDF file)?

The PLOS Data policy requires authors to make all data underlying the findings described in their manuscript fully available without restriction, with rare exception. The data should be provided as part of the manuscript or its supporting information, or deposited to a public repository. For example, in addition to summary statistics, the data points behind means, medians and variance measures should be available. If there are restrictions on publicly sharing data—e.g. participant privacy or use of data from a third party—those must be specified.

Reviewer #1: No

**********

4. Is the manuscript presented in an intelligible fashion and written in standard English?

PLOS Global Public Health does not copyedit accepted manuscripts, so the language in submitted articles must be clear, correct, and unambiguous. Any typographical or grammatical errors should be corrected at revision, so please note any specific errors here.

Reviewer #1: No

**********

5. Review Comments to the Author

Please use the space provided to explain your answers to the questions above. You may also include additional comments for the author, including concerns about dual publication, research ethics, or publication ethics. (Please upload your review as an attachment if it exceeds 20,000 characters)

Reviewer #1: PLOS Global Public Health

ECONOMIC AND HEALTH IMPACTS OF BOVINE TUBERCULOSIS ON RURAL ZAMBIAN COMMUNITIES

General Assessment

This manuscript addresses a relevant and timely topic, exploring the economic and health impacts of bovine tuberculosis (bTB) on rural communities in Zambia through a mixed-methods approach. The work is valuable and provides important insights into the socioeconomic vulnerabilities associated with bTB. However, several areas require clarification and strengthening to enhance the scientific robustness and public health relevance of the study.

Major Comments

1. Missing epidemiological context on zoonotic TB in humans

The manuscript discusses the public health implications of bTB but does not provide available data on M. bovis infection prevalence in humans at:

• national level,

• district level (Lundazi and Monze),

• or from comparable regions in sub-Saharan Africa.

To address this gap, please consider integrating key global references on zoonotic TB, such as:

• WHO (2017). Roadmap for Zoonotic tuberculosis https://www.who.int/publications/i/item/9789241513043

• Olea-Popelka, F., & Fujiwara, P. I. (2018). Building a Multi-Institutional and Interdisciplinary Team to Develop a Zoonotic Tuberculosis Roadmap. Frontiers in Public Health, 6, 167.

https://www.frontiersin.org/articles/10.3389/fpubh.2018.00167/full

Including these references will help contextualize the burden of zoonotic TB and strengthen the public health discussion and better support conclusions.

2. Public health implications are underdeveloped

While the economic impact of bTB is well described, the public health dimension is comparatively limited.

The manuscript would benefit from:

• more explicit discussion of zoonotic risks for different demographic groups,

• potential barriers to diagnosis and reporting of M. bovis in rural healthcare settings,

• implications for One Health surveillance.

This would provide a more balanced interpretation aligned with the study objectives.

3. Limited comparison with existing literature

The discussion currently focuses mainly on East and Southern Africa. It would be helpful to cite global and regional reviews addressing the wildlife–livestock–human interface, which is central to bTB epidemiology in Zambia.

Please consider adding:

De GARINE-WICHATITSKY M, CARON A, KOCK R, et al. 2013 (Cambridge): A review of bovine tuberculosis at the wildlife–livestock–human interface in sub-Saharan Africa https://www.cambridge.org/core/journals/epidemiology-and-infection/article/review-of-bovine-tuberculosis-at-the-wildlifelivestockhuman-interface-in-subsaharan-africa/19D207B4D88531AB03A96FEF7BF6F95E

Munyeme et al. (2011). A Review of Bovine Tuberculosis in the Kafue Basin Ecosystem https://pmc.ncbi.nlm.nih.gov/articles/PMC3087610/

These references are particularly relevant given the role of Kafue lechwe as a reservoir species and the importance of studying disease dynamics at the domestic–wildlife–human interface.

4. Interpretation of increased vulnerability in elderly respondents

The interpretation that elderly individuals are more affected because of reduced immunity and lower awareness requires careful qualification. While it is plausible that older adults may be more likely to progress to clinical disease due to immunosenescence, the study did not include any diagnostic testing for M. bovis infection in humans.

Without diagnostic data, such as tuberculin skin testing (e.g., the Mantoux test), interferon-gamma release assays, or microbiological confirmation, the study cannot infer the true prevalence of mycobacteria infection across age groups.

It is important to acknowledge that younger individuals may have similar or even higher infection rates but remain asymptomatic due to a more effective immune response.

Thus, the distinction between:

• infection prevalence (which requires diagnostic testing), and

• clinical disease expression (more common in immunosuppressed or elderly individuals)

should be clearly stated to avoid overinterpretation of the findings.

5. Methodological clarifications required

Several methodological details require further explanation:

• Clarify whether “strong cough” and “diseased animals” were self-reported or confirmed by veterinary staff.

• Consider discussing potential confounders affecting milk/meat yield (other diseases, nutrition, seasonality).

• Income calculations assume fixed milk prices; please comment on possible seasonal or regional/geographic price variability.

Addressing these points will improve methodological transparency.

Minor Comments

1. Some sections require language editing to improve clarity and flow.

2. Figures and tables would benefit from clearer captions and more detailed descriptions.

3. A brief description of cattle management systems in Lundazi and Monze would provide useful context for interpreting transmission risks.

4. The Discussion could better highlight the value added by the mixed-methods approach.

5. You may consider revising the reference list, as several entries appear duplicated. Specifically, the following references are listed more than once:

• Demetriou 2020 (Refs. 23 and 26)

• Oloya et al. (Refs. 29 and 37)

• Ameni et al. (Refs. 30, 38, and 45)

• Kansiime et al. (Refs. 28 and 36)

• Kazwala et al. (Refs. 32 and 39)

• Mfinanga et al. (Refs. 33 and 40)

• Duguma et al. (Refs. 34 and 41)

• Kassa et al. (Refs. 35 and 42)

Additionally, two reviews by Tschopp (Refs. 18 and 24) appear very similar and may need verification to confirm they are distinct publications.

A careful review of the reference list is recommended to avoid redundancy and improve clarity.

Overall Recommendation

The manuscript provides useful insights but requires substantial revisions. Strengthening the epidemiological context, integrating key references, and refining the public health discussion will significantly improve the impact and clarity of the work and enhance its contribution to PLOS Global Public Health.

**********

6. PLOS authors have the option to publish the peer review history of their article (what does this mean?). If published, this will include your full peer review and any attached files.

**Do you want your identity to be public for this peer review?**     If you choose “no”, your identity will remain anonymous but your review may still be made public.

For information about this choice, including consent withdrawal, please see our Privacy Policy.

Reviewer #1: No

**********

[NOTE: If reviewer comments were submitted as an attachment file, they will be attached to this email and accessible via the submission site. Please log into your account, locate the manuscript record, and check for the action link "View Attachments". If this link does not appear, there are no attachment files.]

-->Figure Resubmissions:

While revising your submission, we strongly recommend that you use PLOS’s NAAS tool (https://ngplosjournals.pagemajik.ai/artanalysis) to test your figure files. NAAS can convert your figure files to the TIFF file type and meet basic requirements (such as print size, resolution), or provide you with a report on issues that do not meet our requirements and that NAAS cannot fix.

After uploading your figures to PLOS’s NAAS tool - https://ngplosjournals.pagemajik.ai/artanalysis, NAAS will process the files provided and display the results in the "Uploaded Files" section of the page as the processing is complete. If the uploaded figures meet our requirements (or NAAS is able to fix the files to meet our requirements), the figure will be marked as "fixed" above. If NAAS is unable to fix the files, a red "failed" label will appear above. When NAAS has confirmed that the figure files meet our requirements, please download the file via the download option, and include these NAAS processed figure files when submitting your revised manuscript.

Attachment Submitted filename: Review Report_PLOS GPH_Zambia bTB_4.12.2025.pdf

# Author comment: Author response to Decision Letter 1

**DOI:** 10.1371/journal.pgph.0005572.r002

11 Feb 2026

Attachment Submitted filename: RESPONSE TO THE REVIEWERS.docx

# Peer review: Decision Letter 1

**DOI:** 10.1371/journal.pgph.0005572.r003

**Authors:** Mavis Pearl Kwabla

4 Mar 2026

PGPH-D-25-03539R1

ECONOMIC AND HEALTH IMPACTS OF BOVINE AND ZOONOTIC TUBERCULOSIS ON RURAL ZAMBIAN COMMUNITIES

PLOS Global Public Health

Dear Dr. Phiri

Thank you for submitting your manuscript to PLOS Global Public Health. After careful consideration, we feel that it has merit but does not fully meet PLOS Global Public Health’s publication criteria as it currently stands. Therefore, we invite you to submit a revised version of the manuscript that addresses the points raised during the review process.

All concerns raised during the initial review have been adequately addressed, with the exception of one issue regarding the details of the analysis performed. Kindly refer to the reviewers’ comments below for further clarification.

Please submit your revised manuscript by March 24, 2026. If you will need more time than this to complete your revisions, please reply to this message or contact the journal office at globalpubhealth@plos.org. When you're ready to submit your revision, log on to https://www.editorialmanager.com/pgph/ and select the 'Submissions Needing Revision' folder to locate your manuscript file.

Please include the following items when submitting your revised manuscript:

- A letter that responds to each point raised by the editor and reviewer(s). You should upload this letter as a separate file labeled 'Response to Reviewers'.
- A marked-up copy of your manuscript that highlights changes made to the original version. You should upload this as a separate file labeled 'Revised Manuscript with Track Changes'.
- An unmarked version of your revised paper without tracked changes. You should upload this as a separate file labeled 'Manuscript'.

Guidelines for resubmitting your figure files are available below the reviewer comments at the end of this letter.

We look forward to receiving your revised manuscript.

Kind regards,

Mavis Pearl Kwabla, Master of Public Health

Academic Editor

PLOS Global Public Health

Journal Requirements:

If the reviewer comments include a recommendation to cite specific previously published works, please review and evaluate these publications to determine whether they are relevant and should be cited. There is no requirement to cite these works unless the editor has indicated otherwise.

Please review your reference list to ensure that it is complete and correct. If you have cited papers that have been retracted, please include the rationale for doing so in the manuscript text, or remove these references and replace them with relevant current references. Any changes to the reference list should be mentioned in the rebuttal letter that accompanies your revised manuscript. If you need to cite a retracted article, indicate the article’s retracted status in the References list and also include a citation and full reference for the retraction notice.

Additional Editor Comments (if provided):

Dear Authors,

The reviewers have confirmed that you have adequately addressed the main comments raised during the previous round of review and have revised the manuscript accordingly. However, the concern regarding the limited information provided about the data analysis remains. Specifically, details of the descriptive and inferential statistical methods are insufficient. For example, the manuscript does not clearly state the statistical modeling approach used to assess the association between bTB impact and monthly income.

We recommend that you address this issue to improve the methodological clarity and transparency of the manuscript. Accordingly, the manuscript requires minor revisions before it can be considered for publication in PLOS Global Public Health.

Kind regards,

Dr. Mavis Pearl Kwabla (PhD, MPH)

Academic Editor

[Note: HTML markup is below. Please do not edit.]

Reviewers' comments:

Reviewer's Responses to Questions

**Comments to the Author**

1. If the authors have adequately addressed your comments raised in a previous round of review and you feel that this manuscript is now acceptable for publication, you may indicate that here to bypass the “Comments to the Author” section, enter your conflict of interest statement in the “Confidential to Editor” section, and submit your "Accept" recommendation.

Reviewer #1: All comments have been addressed

**********

2. Does this manuscript meet PLOS Global Public Health’s publication criteria? Is the manuscript technically sound, and do the data support the conclusions? The manuscript must describe methodologically and ethically rigorous research with conclusions that are appropriately drawn based on the data presented.

Reviewer #1: Partly

**********

3. Has the statistical analysis been performed appropriately and rigorously?

Reviewer #1: No

**********

4. Have the authors made all data underlying the findings in their manuscript fully available (please refer to the Data Availability Statement at the start of the manuscript PDF file)?

The PLOS Data policy requires authors to make all data underlying the findings described in their manuscript fully available without restriction, with rare exception. The data should be provided as part of the manuscript or its supporting information, or deposited to a public repository. For example, in addition to summary statistics, the data points behind means, medians and variance measures should be available. If there are restrictions on publicly sharing data—e.g. participant privacy or use of data from a third party—those must be specified.

Reviewer #1: No

**********

5. Is the manuscript presented in an intelligible fashion and written in standard English?

PLOS Global Public Health does not copyedit accepted manuscripts, so the language in submitted articles must be clear, correct, and unambiguous. Any typographical or grammatical errors should be corrected at revision, so please note any specific errors here.

Reviewer #1: No

**********

6. Review Comments to the Author

Please use the space provided to explain your answers to the questions above. You may also include additional comments for the author, including concerns about dual publication, research ethics, or publication ethics. (Please upload your review as an attachment if it exceeds 20,000 characters)

Reviewer #1: The authors have addressed the main comments raised during the previous review round and have revised the manuscript accordingly. The requested references have been incorporated, and additional clarifications have been provided in the methodological and discussion sections. The manuscript has improved in structure and contextualization.

One point that would benefit from further clarification concerns the statistical modeling approach used to assess the association between bTB impact and monthly income.

While the manuscript has been substantially revised, the statistical modeling approach used to assess the association between bTB impact and monthly income remains insufficiently described. Clarification of the model specification (type of regression, outcome scaling, and interpretation of the reported odds ratio) would improve transparency. In particular, the reporting of an odds ratio (OR = 0.99) for income expressed in ZMW, together with a confidence interval of 0.997–0.998, would benefit from clearer explanation regarding model structure and variable scaling.

This appears primarily to be an issue of reporting and transparency rather than a fundamental flaw in the analysis. Addressing this point would strengthen the methodological clarity of the manuscript.

**********

7. PLOS authors have the option to publish the peer review history of their article (what does this mean?). If published, this will include your full peer review and any attached files.

**Do you want your identity to be public for this peer review?**     If you choose “no”, your identity will remain anonymous but your review may still be made public.

For information about this choice, including consent withdrawal, please see our Privacy Policy.

Reviewer #1: No

**********

[NOTE: If reviewer comments were submitted as an attachment file, they will be attached to this email and accessible via the submission site. Please log into your account, locate the manuscript record, and check for the action link "View Attachments". If this link does not appear, there are no attachment files.]

Figure Resubmissions:

While revising your submission, we strongly recommend that you use PLOS’s NAAS tool (https://ngplosjournals.pagemajik.ai/artanalysis) to test your figure files. NAAS can convert your figure files to the TIFF file type and meet basic requirements (such as print size, resolution), or provide you with a report on issues that do not meet our requirements and that NAAS cannot fix.

After uploading your figures to PLOS’s NAAS tool - https://ngplosjournals.pagemajik.ai/artanalysis, NAAS will process the files provided and display the results in the "Uploaded Files" section of the page as the processing is complete. If the uploaded figures meet our requirements (or NAAS is able to fix the files to meet our requirements), the figure will be marked as "fixed" above. If NAAS is unable to fix the files, a red "failed" label will appear above. When NAAS has confirmed that the figure files meet our requirements, please download the file via the download option, and include these NAAS processed figure files when submitting your revised manuscript.

# Author comment: Author response to Decision Letter 2

**DOI:** 10.1371/journal.pgph.0005572.r004

4 Mar 2026

Attachment Submitted filename: RESPONSE_TO_THE_REVIEWERS_auresp_2.docx

# Peer review: Decision Letter 2

**DOI:** 10.1371/journal.pgph.0005572.r005

**Authors:** Mavis Pearl Kwabla

17 Mar 2026

PGPH-D-25-03539R2

ECONOMIC AND HEALTH IMPACTS OF BOVINE AND ZOONOTIC TUBERCULOSIS ON RURAL ZAMBIAN COMMUNITIES

PLOS Global Public Health

Dear Dr. Phiri Anthony,

Thank you for submitting your manuscript to PLOS Global Public Health. After careful consideration, we feel that it has merit but does not fully meet PLOS Global Public Health’s publication criteria as it currently stands. Therefore, we invite you to submit a revised version of the manuscript that addresses the points raised during the review process.

Kindly check the reviewers comments for further details.

Please submit your revised manuscript by 31st March, 2026. If you will need more time than this to complete your revisions, please reply to this message or contact the journal office at globalpubhealth@plos.org. When you're ready to submit your revision, log on to https://www.editorialmanager.com/pgph/ and select the 'Submissions Needing Revision' folder to locate your manuscript file.

Please include the following items when submitting your revised manuscript:

- A letter that responds to each point raised by the editor and reviewer(s). You should upload this letter as a separate file labeled 'Response to Reviewers'.
- A marked-up copy of your manuscript that highlights changes made to the original version. You should upload this as a separate file labeled 'Revised Manuscript with Track Changes'.
- An unmarked version of your revised paper without tracked changes. You should upload this as a separate file labeled 'Manuscript'.

Guidelines for resubmitting your figure files are available below the reviewer comments at the end of this letter.

We look forward to receiving your revised manuscript.

Kind regards,

Mavis Pearl Kwabla, Master of Public Health

Academic Editor

PLOS Global Public Health

**Journal Requirements:**

If the reviewer comments include a recommendation to cite specific previously published works, please review and evaluate these publications to determine whether they are relevant and should be cited. There is no requirement to cite these works unless the editor has indicated otherwise.

Please review your reference list to ensure that it is complete and correct. If you have cited papers that have been retracted, please include the rationale for doing so in the manuscript text, or remove these references and replace them with relevant current references. Any changes to the reference list should be mentioned in the rebuttal letter that accompanies your revised manuscript. If you need to cite a retracted article, indicate the article’s retracted status in the References list and also include a citation and full reference for the retraction notice.

**Additional Editor Comments (if provided):**

Dear Authors,

Thank you for your efforts in revising the manuscript and for addressing the major concerns raised in the previous rounds of review. The manuscript has improved substantially. However, a few issues remain that need to be addressed before it can be considered for publication.

First, the statistical analysis requires further clarification. The use of ordinal logistic regression is not clearly justified. The outcome variable has not been defined as ordinal, and the reported odds ratios (ORs) and confidence intervals appear inconsistent. Please clearly state the outcome variable used in the ordinal logistic regression and explain how it was categorized.

Second, some subheadings in the methods section overlap and should be reorganized to improve clarity and reduce redundancy. The subsection on the “Impact of bTB on Monthly Income” would be more appropriately included under the Data Analysis section. In addition, please revise “Data analysis tools” to “Data analysis.”

Third, the Results section of the abstract (quantitative component) should include key statistical details. Statements indicating strong associations, for example, between bTB impact and decreased income, should report the corresponding ORs and confidence intervals. Similarly, findings such as the effect of primary education on bTB awareness or transmission should be supported with appropriate statistical estimates.

Addressing these issues will strengthen the clarity and rigor of the manuscript. I look forward to reviewing a revised version.

Kind regards,

Dr. Mavis Pearl Kwabla (PhD, MPH)

Academic Editor

[Note: HTML markup is below. Please do not edit.]

Reviewers' comments:

Reviewer's Responses to Questions

**Comments to the Author**

1. If the authors have adequately addressed your comments raised in a previous round of review and you feel that this manuscript is now acceptable for publication, you may indicate that here to bypass the “Comments to the Author” section, enter your conflict of interest statement in the “Confidential to Editor” section, and submit your "Accept" recommendation.

Reviewer #1: All comments have been addressed

**********

2. Does this manuscript meet PLOS Global Public Health’s publication criteria? Is the manuscript technically sound, and do the data support the conclusions? The manuscript must describe methodologically and ethically rigorous research with conclusions that are appropriately drawn based on the data presented.

Reviewer #1: Partly

**********

3. Has the statistical analysis been performed appropriately and rigorously?

Reviewer #1: No

**********

4. Have the authors made all data underlying the findings in their manuscript fully available (please refer to the Data Availability Statement at the start of the manuscript PDF file)?

The PLOS Data policy requires authors to make all data underlying the findings described in their manuscript fully available without restriction, with rare exception. The data should be provided as part of the manuscript or its supporting information, or deposited to a public repository. For example, in addition to summary statistics, the data points behind means, medians and variance measures should be available. If there are restrictions on publicly sharing data—e.g. participant privacy or use of data from a third party—those must be specified.

Reviewer #1: No

**********

5. Is the manuscript presented in an intelligible fashion and written in standard English?

PLOS Global Public Health does not copyedit accepted manuscripts, so the language in submitted articles must be clear, correct, and unambiguous. Any typographical or grammatical errors should be corrected at revision, so please note any specific errors here.

Reviewer #1: No

**********

6. Review Comments to the Author

Please use the space provided to explain your answers to the questions above. You may also include additional comments for the author, including concerns about dual publication, research ethics, or publication ethics. (Please upload your review as an attachment if it exceeds 20,000 characters)

**Reviewer #1:**    PLOS Global Public Health

ECONOMIC AND HEALTH IMPACTS OF BOVINE TUBERCULOSIS ON RURAL ZAMBIAN COMMUNITIES

1. Major comment – Case definition of bovine tuberculosis

As already noted in my previous report, the manuscript discusses the impact of bovine tuberculosis (bTB) on cattle production and household income. Following the clarification now included in the Methods section, the presence of bTB in cattle appears to be based on self-reported symptoms (e.g., “strong cough”) provided by farmers and other participants, without veterinary confirmation or diagnostic testing. Since respiratory symptoms alone are not specific to bTB, this approach may lead to potential misclassification of cases. I therefore recommend clarifying throughout the manuscript that the study refers to reported disease signs potentially compatible with bTB, rather than confirmed bTB infections, to avoid possible over-interpretation of the findings.

In addition, the Results section reports that 50 out of 280 cattle (17.9%) showed signs of “strong cough”, which is interpreted as evidence of bTB impact. Given that this estimate is based on self-reported symptoms rather than diagnostic confirmation, this figure should be interpreted more cautiously. It would be helpful to clarify that this proportion reflects reported respiratory symptoms potentially compatible with disease, and therefore the perceived presence and impact of disease among farmers, rather than confirmed bTB prevalence, which would strengthen the epidemiological interpretation of the results.

2. Major comment – Statistical modelling

The description and interpretation of the ordinal logistic regression model require further clarification. In particular, the manuscript does not clearly define the ordinal outcome variable used in the model, nor how monthly income was categorized to justify the use of an ordinal regression approach. In addition, the reported regression results appear inconsistent (e.g., OR = 0.99 with CI = 0.997–0.998), and the interpretation of the odds ratio is difficult to follow. I recommend verifying the regression outputs and clearly describing the model specification, variables included, and the interpretation of the results. In addition, since ordinal logistic regression relies on the proportional odds assumption, it would be useful to indicate whether this assumption was assessed. A careful review of the statistical analysis would help ensure that the model specification and interpretation are appropriate.

3. Minor Comment – Methods structure

The Methods section has been expanded in the revised manuscript, which improves transparency. However, some subsections partially overlap (e.g., Sample size, Selection of participants, and Study population and sampling strategy). Consider reorganizing these sections to improve readability and reduce redundancy.

4. Minor Comment – Economic loss estimates

The description of milk production losses requires clarification. The text indicates that a diseased cow shows “a reduction in milk production of close to 3.75 liters”, whereas Table 2 suggests that milk production decreases from 5 L to 3.75 L (i.e., a reduction of 1.25 L). Please ensure consistency between the text and the table and clarify the calculation of the reported economic losses.

5. Minor Comment – Language and typographical issues

The manuscript is generally understandable; however, some minor language issues and typographical errors remain (e.g., “These areas boast of substantial cattle populations”, “very much recommended time frame”, and “1medical practitioners”). A careful language editing would improve clarity and overall readability.

**********

7. PLOS authors have the option to publish the peer review history of their article (what does this mean?). If published, this will include your full peer review and any attached files.

**Do you want your identity to be public for this peer review?**     If you choose “no”, your identity will remain anonymous but your review may still be made public.

For information about this choice, including consent withdrawal, please see our Privacy Policy.

Reviewer #1: No

**********

[NOTE: If reviewer comments were submitted as an attachment file, they will be attached to this email and accessible via the submission site. Please log into your account, locate the manuscript record, and check for the action link "View Attachments". If this link does not appear, there are no attachment files.]

Figure Resubmissions:

While revising your submission, we strongly recommend that you use PLOS’s NAAS tool (https://ngplosjournals.pagemajik.ai/artanalysis) to test your figure files. NAAS can convert your figure files to the TIFF file type and meet basic requirements (such as print size, resolution), or provide you with a report on issues that do not meet our requirements and that NAAS cannot fix.

After uploading your figures to PLOS’s NAAS tool - https://ngplosjournals.pagemajik.ai/artanalysis, NAAS will process the files provided and display the results in the "Uploaded Files" section of the page as the processing is complete. If the uploaded figures meet our requirements (or NAAS is able to fix the files to meet our requirements), the figure will be marked as "fixed" above. If NAAS is unable to fix the files, a red "failed" label will appear above. When NAAS has confirmed that the figure files meet our requirements, please download the file via the download option, and include these NAAS processed figure files when submitting your revised manuscript.

Attachment Submitted filename: PLOS GPH_Report 3.docx.pdf

# Author comment: Author response to Decision Letter 3

**DOI:** 10.1371/journal.pgph.0005572.r006

19 Mar 2026

Attachment Submitted filename: RESPONSE_TO_THE_REVIEWERS_auresp_3.docx

# Peer review: Decision Letter 3

**DOI:** 10.1371/journal.pgph.0005572.r007

**Authors:** Mavis Pearl Kwabla

22 Apr 2026

PGPH-D-25-03539R3

ECONOMIC AND HEALTH IMPACTS OF BOVINE AND ZOONOTIC TUBERCULOSIS ON RURAL ZAMBIAN COMMUNITIES

PLOS Global Public Health

Dear Dr. Phiri Anthony,

Thank you for submitting your manuscript to PLOS Global Public Health. After careful consideration, we feel that it has merit but does not fully meet PLOS Global Public Health’s publication criteria as it currently stands. Therefore, we invite you to submit a revised version of the manuscript that addresses the points raised during the review process.

Please submit your revised manuscript by April 28, 2026. If you will need more time than this to complete your revisions, please reply to this message or contact the journal office at globalpubhealth@plos.org. When you're ready to submit your revision, log on to https://www.editorialmanager.com/pgph/ and select the 'Submissions Needing Revision' folder to locate your manuscript file.

Please include the following items when submitting your revised manuscript:

- A letter that responds to each point raised by the editor and reviewer(s). You should upload this letter as a separate file labeled 'Response to Reviewers'.
- A marked-up copy of your manuscript that highlights changes made to the original version. You should upload this as a separate file labeled 'Revised Manuscript with Track Changes'.
- An unmarked version of your revised paper without tracked changes. You should upload this as a separate file labeled 'Manuscript'.

Guidelines for resubmitting your figure files are available below the reviewer comments at the end of this letter.

As the corresponding author, your ORCID iD is verified in the submission system and will appear in the published article. PLOS supports the use of ORCID, and we encourage all coauthors to register for an ORCID iD and use it as well. Please encourage your coauthors to verify their ORCID iD within the submission system before final acceptance, as unverified ORCID iDs will not appear in the published article. *Only*     the individual author can complete the verification step; PLOS staff *cannot*     verify ORCID iDs on behalf of authors.

We look forward to receiving your revised manuscript.

Kind regards,

Mavis Pearl Kwabla, Master of Public Health

Academic Editor

PLOS Global Public Health

**Journal Requirements:**

If the reviewer comments include a recommendation to cite specific previously published works, please review and evaluate these publications to determine whether they are relevant and should be cited. There is no requirement to cite these works unless the editor has indicated otherwise.

Please review your reference list to ensure that it is complete and correct. If you have cited papers that have been retracted, please include the rationale for doing so in the manuscript text, or remove these references and replace them with relevant current references. Any changes to the reference list should be mentioned in the rebuttal letter that accompanies your revised manuscript. If you need to cite a retracted article, indicate the article’s retracted status in the References list and also include a citation and full reference for the retraction notice.

**Additional Editor Comments (if provided):**

Dear Authors,

Thank you for submitting the revised version of your manuscript and for your thoughtful responses to the reviewers’ comments.

The manuscript has improved considerably following the previous round of revisions. In particular, the clarification regarding the classification of cattle disease status, which is based on reported symptoms compatible with bovine tuberculosis (bTB), as well as the additional details provided on the ordinal logistic regression model, has strengthened the methodological rigor and clarity of the study.

After careful consideration of the reviewers’ comments, I wish to inform you that your manuscript is recommended to undergo minor revisions, after which it can be considered for publication.

The remaining issues to address are as follows:

1. Statistical reporting

Please explicitly report the income categories (including cut-off values) used to define the ordered outcome variable in the ordinal logistic regression model. This will improve transparency and reproducibility.

2. Language and consistency

A final round of careful language editing is needed to address minor grammatical issues and ensure consistency in terminology (e.g., consistent use of “reported bTB-like symptoms” where appropriate, and removal of duplicated section headings).

3. Discussion section clarity

The discussion should be revised to more clearly distinguish findings from your study from those drawn from existing literature. At present, many points are heavily referenced, making it difficult to identify your study’s specific contributions.

In addition, detailed comments have been provided in the tracked changes within the manuscript to guide your revisions.

We believe that addressing these minor issues will further strengthen your manuscript and make it suitable for publication.

We look forward to receiving your revised manuscript.

Kind regards,

Dr. Mavis Pearl Kwabla (PhD, MPH)

Academic Editor

[Note: HTML markup is below. Please do not edit.]

Reviewers' comments:

Reviewer's Responses to Questions

**Comments to the Author**

1. If the authors have adequately addressed your comments raised in a previous round of review and you feel that this manuscript is now acceptable for publication, you may indicate that here to bypass the “Comments to the Author” section, enter your conflict of interest statement in the “Confidential to Editor” section, and submit your "Accept" recommendation.

Reviewer #1: All comments have been addressed

**********

2. Does this manuscript meet PLOS Global Public Health’s publication criteria? Is the manuscript technically sound, and do the data support the conclusions? The manuscript must describe methodologically and ethically rigorous research with conclusions that are appropriately drawn based on the data presented.

Reviewer #1: Partly

**********

3. Has the statistical analysis been performed appropriately and rigorously?

Reviewer #1: No

**********

4. Have the authors made all data underlying the findings in their manuscript fully available (please refer to the Data Availability Statement at the start of the manuscript PDF file)?

The PLOS Data policy requires authors to make all data underlying the findings described in their manuscript fully available without restriction, with rare exception. The data should be provided as part of the manuscript or its supporting information, or deposited to a public repository. For example, in addition to summary statistics, the data points behind means, medians and variance measures should be available. If there are restrictions on publicly sharing data—e.g. participant privacy or use of data from a third party—those must be specified.

Reviewer #1: No

**********

5. Is the manuscript presented in an intelligible fashion and written in standard English?

PLOS Global Public Health does not copyedit accepted manuscripts, so the language in submitted articles must be clear, correct, and unambiguous. Any typographical or grammatical errors should be corrected at revision, so please note any specific errors here.

Reviewer #1: No

**********

6. Review Comments to the Author

Please use the space provided to explain your answers to the questions above. You may also include additional comments for the author, including concerns about dual publication, research ethics, or publication ethics. (Please upload your review as an attachment if it exceeds 20,000 characters)

**Reviewer #1:**    PLOS Global Public Health

ECONOMIC AND HEALTH IMPACTS OF BOVINE TUBERCULOSIS ON RURAL ZAMBIAN COMMUNITIES

General comment

The revised manuscript has improved substantially in response to the previous comments. In particular, the clarification that cattle disease status was based on reported symptoms potentially compatible with bTB rather than confirmed infection, together with the additional information provided on the ordinal logistic regression model, represents a clear improvement. However, a few issues remain, including clearer reporting of the income categories used in the model, consistency of terminology across sections, and minor language editing.

Remaining comment 1 – Statistical reporting

While the regression model is now better described, it would still be helpful to explicitly report the income categories (cut-off values) used to define the ordered outcome variable in the ordinal logistic regression model, in order to improve transparency and reproducibility.

Remaining comment 2 – Language and consistency

A few minor language issues and inconsistencies in terminology remain (e.g., occasional use of “bTB” where “reported bTB-like symptoms” may be more accurate, duplicated section headings, and minor grammatical phrasing). A final careful language editing would further improve readability.

Overall, the manuscript has improved considerably and, after minor revisions, may be suitable for publication.

**********

7. PLOS authors have the option to publish the peer review history of their article (what does this mean?). If published, this will include your full peer review and any attached files.

**Do you want your identity to be public for this peer review?**     If you choose “no”, your identity will remain anonymous but your review may still be made public.

For information about this choice, including consent withdrawal, please see our Privacy Policy.

Reviewer #1: No

**********

[NOTE: If reviewer comments were submitted as an attachment file, they will be attached to this email and accessible via the submission site. Please log into your account, locate the manuscript record, and check for the action link "View Attachments". If this link does not appear, there are no attachment files.]

**Figure Resubmissions:**

While revising your submission, we strongly recommend that you use PLOS’s NAAS tool (https://ngplosjournals.pagemajik.ai/artanalysis) to test your figure files. NAAS can convert your figure files to the TIFF file type and meet basic requirements (such as print size, resolution), or provide you with a report on issues that do not meet our requirements and that NAAS cannot fix.

After uploading your figures to PLOS’s NAAS tool - https://ngplosjournals.pagemajik.ai/artanalysis, NAAS will process the files provided and display the results in the "Uploaded Files" section of the page as the processing is complete. If the uploaded figures meet our requirements (or NAAS is able to fix the files to meet our requirements), the figure will be marked as "fixed" above. If NAAS is unable to fix the files, a red "failed" label will appear above. When NAAS has confirmed that the figure files meet our requirements, please download the file via the download option, and include these NAAS processed figure files when submitting your revised manuscript.

# Author comment: Author response to Decision Letter 4

**DOI:** 10.1371/journal.pgph.0005572.r008

22 Apr 2026

Attachment Submitted filename: REBUTTAL LETTER 22APRIL26.docx

# Editor report: Decision Letter 4

**DOI:** 10.1371/journal.pgph.0005572.r009

**Authors:** Mavis Pearl Kwabla

30 Apr 2026

ECONOMIC AND HEALTH IMPACTS OF BOVINE AND ZOONOTIC TUBERCULOSIS ON RURAL ZAMBIAN COMMUNITIES

PGPH-D-25-03539R4

Dear Dr. Phiri Anthony,

We are pleased to inform you that your manuscript 'ECONOMIC AND HEALTH IMPACTS OF BOVINE AND ZOONOTIC TUBERCULOSIS ON RURAL ZAMBIAN COMMUNITIES' has been provisionally accepted for publication in PLOS Global Public Health.

Before your manuscript can be formally accepted you will need to complete some formatting changes, which you will receive in a follow up email. A member of our team will be in touch with a set of requests.

Please note that your manuscript will not be scheduled for publication until you have made the required changes, so a swift response is appreciated.

IMPORTANT: The editorial review process is now complete. PLOS will only permit corrections to spelling, formatting or significant scientific errors from this point onwards. Requests for major changes, or any which affect the scientific understanding of your work, will cause delays to the publication date of your manuscript.

If your institution or institutions have a press office, please notify them about your upcoming paper to help maximize its impact. If they'll be preparing press materials, please inform our press team as soon as possible -- no later than 48 hours after receiving the formal acceptance. Your manuscript will remain under strict press embargo until 2 pm Eastern Time on the date of publication. For more information, please contact globalpubhealth@plos.org.

Thank you again for supporting Open Access publishing; we are looking forward to publishing your work in PLOS Global Public Health.

Best regards,

Mavis Pearl Kwabla, Master of Public Health

Academic Editor

PLOS Global Public Health

***********************************************************

Dear Authors,

Thank you for submitting the revised version of your manuscript. I have carefully reviewed your responses to the reviewers’ comments, as well as the updated manuscript.

I am pleased to note that the manuscript has improved substantially following the previous round of review. The key issues raised earlier have been satisfactorily addressed. In particular, the clarification that cattle disease status was based on reported symptoms compatible with bovine tuberculosis (bTB), rather than confirmed infection, significantly strengthens the interpretation of your findings. Additionally, the expanded description of the ordinal logistic regression model has improved the transparency and rigor of your methodological approach.

The reporting of income categories and their corresponding cut-off values is now clear, and the revisions to the discussion section better distinguish your study’s findings from those of other studies.

Only a few minor issues are remaining, mainly typographical and formatting errors (for example, the discussion section is currently labeled as “Conclusion,” along with a few other minor inconsistencies). These can be addressed during the final stages of editorial processing.

Based on the revisions made and the current quality of the manuscript, I am pleased to inform you that your manuscript is recommended for acceptance, subject to minor editorial corrections.

Congratulations, and thank you for your careful and thorough revisions.

Kind regards,

Dr. Mavis Pearl Kwabla (PhD, MPH)

Academic Editor

Reviewer Comments (if any, and for reference):
